# 

**Published:** 1914-03-14

**Authors:** 


### A HOSPITAL WARD ON THE STAGE.

A play entitled "A Powerful Remedy," by Kerry Gordon, touching upon the hereditary craving for alcohol, will be performed at a special matinée at the London Pavilion on March 25 at 2.30 p.m. The scene is a side ward in a hospital. The leading characters are an Irish journalist and a probationer. The author, who, it is stated, trained as a nurse in Dublin, is the wife of Dr. Laing Gordon, author of "The Life of Sir J. Y. Simpson." Tickets, ranging from 7s. 6d. to 2s. reserved, may be obtained from Department G, West End Productions, Ltd.

### PRUDENTIAL ASSURANCE COMPANY.

The report and accounts of this company for the year 1913 show that the number of policies issued during the year was 71,359, assuring the sum of £6,849,224, and producing a new annual premium income of £425,717. The premiums received during the year were £4,920,518, being an increase of £93,525 over the year 1912. In addition, £11,116 was received in premiums under the Sickness Insurance Tables. The claims of the year amounted to £3,766,625. The number of deaths was 8,699. The number of endowment assurances matured was 23,497, the premium income of which was £131,017. The number of policies in force at the end of the year was 917,091.

Industrial Branch.—The premiums received during the year were £7,874,456, being an increase of £81,894. The increase shown would have been much greater but for the fact that, owing to the system of accounts, fifty-three weekly collections were credited in the report for the year 1912. The claims of the year amounted to £3,139,193, including 359,572 bonus additions. The number of claims and surrenders, including 5,942 endowment assurances matured, was 366,104. The assets of the company in both branches, as shown in the balance-sheet, after deducting £1,750,000 written off securities, are £86,993,003, being an increase of £2,421,071 over those of 1912.

### Appointments.

Mr. J. H. Madill has been appointed sixth assistant medical officer at Hanwell Mental Hospital.

Mr. W. Moodie has been promoted to be fourth assistant medical officer at Claybury Mental Hospital, and Mr. H. M. Rashbrook and Mr. F. H. Guppy have been appointed as fifth and sixth assistants respectively.

Dr. V. Glendining, M.B., B.S., of Guy's Hospital, has been appointed by the Southwark Board of Guardians temporary medical officer at their infirmary, at a salary of £5 5s. a week, in place of Dr. R. A. Banbury, who has resigned.

Dr. C. H. McGuire and Dr. A. W. Russell have been appointed indoor house surgeons to the Glasgow Maternity and Women's Hospital for six months from April 4 prox.

Mr. J. W. Pugh, M.D., M.R.C.S., Mr. J. R. Higson, M.B., C.M., D.P.H., and Mr. Julian Landman, M.D., Ch.B., have been appointed clinical assistants to the West End Hospital.

To the post of tuberculosis officer for Berkshire has been elected Dr. Arthur Richmond, M.R.C.S., L.R.C.P., D.P.H.(Camb.), who comes from doing similar work in the West Riding of Yorkshire. Dr. Richmond was formerly resident medical officer at the Birmingham General Hospital, and has held public health appointments in Essex.

Dr. Arthur Herbert Norris, M.R.C.S., L.R.C.P., D.P.H.(Vict.), has been appointed to an inspectorship under the Home Office in connection with the control of industrial schools. Dr. Norris will shortly relinquish the post of tuberculosis officer to the Berkshire County Council and medical adviser to the Berkshire Insurance Committee. He was formerly one of the school medical officers for the same county, and medical officer at the Manchester Children's Hospital.

Mr. Harold Turley Mant has recently been appointed laryngologist to the Royal Hospital for Diseases of the Chest, City Road, E.C. Mr. Mant, who received his medical education at University College Hospital, holds the M.S. of the London University and the Fellowship of the Royal College of Surgeons, England. He is surgeon to the ear and throat department of the Great Northern Central Hospital and surgical registrar at the Golden Square Throat Hospital. Another newly created post at the City Road Chest Hospital is that of cardiologist. Dr. Arnold Walmsley Stott, M.R.C.P., was appointed a few months ago. He is a "Bart.'s" man, and is the chief assistant in the children's department, and demonstrator of pathology at St. Bartholomew's Hospital.

The post of clerk and collector to the Monkwearmouth and Southwick Hospital, referred to in our columns last week, is held by Mr. W. E. Mann.

Vacancies for the post of medical registrar and for various house appointments occur at the Seamen's Hospital, full particulars of which will be found in our advertisement columns.

### Gifts and Bequests.

LORD MAYOR TRELOAR CRIPPLES' HOSPITAL, Alton, has received £1,000 from Mr. MacGarvey, of Vienna.

THE choristers of Westminster Cathedral are giving in the Cathedral Hall a series of performances of "Everyman," the proceeds of which are to be given to St. Andrew's Hospital, Dollis Hill.

MR. CHARLES BRUCE, LL.D., banker, of Edinburgh, has left immediate and conditional bequests to charity, including £3,500 each to the Royal Infirmary and the Royal Hospital for Incurables, Edinburgh.

MR. GEORGE DAY, of Creech St. Michael, Somerset, cattle dealer, subject to his wife's life interest, has left the Heydon House Farm, of about 117 acres, near Taunton, upon trust to apply the income towards the current expenses of the Taunton Hospital.

MAJOR GEORGE HORATIO BRAND, of Portman Square, has left £1,000 to St. Mary's Hospital, Paddington, for a "Major George Horatio Brand" Bed, and among other charitable bequests one of £5 to "William Smith, crossing-sweeper with frozen feet, at the corner of Portman Square."

LORD DONOUGHMORE, treasurer of the London Homœopathic Hospital, has received the legacy of £5,000 bequeathed by the late Mrs. F. G. Smart, of Tunbridge Wells. Mrs. Smart was the widow of Dr. Smart, who for many years had a seat on the board of management of the hospital.

MR. JAMES FERGUSON POWER, lead and glass manufacturer and looking-glass maker, has bequeathed £2,000 to St. Mary's Hospital and the Manchester and Salford Lying-in Hospital and Dispensary, £1,000 to the Manchester Infirmary, Dispensary, and Lunatic Hospital or Asylum, and £500 to the Manchester and Salford Blind Aid Society.

MRS. SARAH CAMPBELL, of Shepherd's Bush, has bequeathed £100 to Dr. Barnardo's Homes, £50 to Nazareth House, Hammersmith, £1,000 each to the Cancer Hospital, Brompton, the Brompton Hospital for Diseases of the Chest, West London Hospital, and the London Hospital; £500 each to the Royal Hospital for Incurables, Middlesex Hospital, and Sir William Treloar's Cripple Fund, the bequests to the said hospitals to be for the endowment of "Sarah Campbell" Beds.

MR. JOHN SHIEL, of Newcastle-upon-Tyne, chairman of the South Garesfield Colliery Company (Limited) and of the Lintz Colliery, and a director of the Framwellgate Coal and Coke Company, left estate of the gross value of £67,269, of which the net personalty has been sworn at £54,606. The testator left by his will a legacy of £1,000 to the Harrogate Convalescent Home, in connection with the Durham County Hospital, but by codicil he revoked this bequest "in view of the provisions under the Insurance Bill and sanatoria provided thereby."

### A GRACEFUL THANKOFFERING.

THE Board of Management of King Edward VII.'s Hospital, Cardiff, has received a donation of 100 guineas from Mr. W. H. Seager as a thankoffering on the occasion of one of his sons attaining his majority.

### GIFT TO LEIGH INFIRMARY.

MRS. GREENOUGH and her son, Mr. T. Greenough, of Beechwood, have presented to Leigh Infirmary, in memory of the late Alderman Greenough, a block of buildings in which to house a complete installation of $x$-ray apparatus.

### The Book Market.

LIST OF NEW BOOKS RECEIVED FROM THE FOLLOWING PUBLISHERS:—

E. Arnold: "The Practice of Surgery." By Russell Howard.
H. K. Lewis: "Health." By M. M. Burgess.
G. Routledge and Sons, Ltd.: "Women Workers in Seven Professions." By Edith J. Morley.
Simpkin, Marshall: "Carbon Dioxide Snow." By J. Hall-Edwards.

**Institution and Public Reports.**

Fourth Annual Report (1913) of the Women's Imperial Health Association, 7 Hanover Square, W.
Report for 1913 of the Clapham General and Provident Dispensary, Manor Street, S.W.
Tenth Annual Report, for 1913, of the Pontypool and District Hospital.
The Report of the Royal Devon and Exeter Hospital.
The 113th Annual Report of the Aberdeen Royal Asylum.

BIG BEQUESTS OF A REDHILL LADY.

Some munificent bequests are contained in the will of Mrs. Marianne Costar, of Redhill, whose estate runs into six figures. She has left £5,000 each to St. Thomas's Hospital, Guy's Hospital, and the Reedham Orphanage. Twelve more institutions receive £2,000 apiece: these are, with one exception, Metropolitan hospitals; and the list includes the London Hospital, Charing Cross Hospital, the Great Northern Hospital, Middlesex Hospital, the Metropolitan Hospital, King's College Hospital, St. Mary's Hospital, University College Hospital, the Westminster Hospital, the Royal Free Hospital, the West London Hospital, and the Redhill and Reigate Cottage Hospital. Compared with some of the vast bequests to individual charities which have been announced within the last twelve-month, these sums in themselves may appear moderate, but the total thus allotted to charities comes to almost £40,000, a generous proportion out of a fortune of £125,000. We offer our congratulations to the institutions concerned.

RATES OF SUBSCRIPTION: Twelve months, 6/6; Six months, 4/-; Three months, 2/-, post free to any address in the United Kingdom. Abroad, Twelve months, 8/8; Six months, 5/-; Three months, 2/6, post free.—Address The Subscription Dept., "THE HOSPITAL," The Hospital Building, 28/29 Southampton Street, Strand, London, W.C.

